# Supplementary material for: Microbial context predicts SARS-CoV-2 prevalence in patients and the hospital built environment
Source: medRxiv. 2020 Nov 22:2020.11.19.20234229. Preprint. [Version 1] doi: 10.1101/2020.11.19.20234229 (PMC7685343; doi:10.1101/2020.11.19.20234229)
Supplement: 1 [file NIHPP2020.11.19.20234229-supplement-1.pdf]

medRxiv preprint doi: https://doi.org/10.1101/2020.11.19.20234229; this version posted November 22, 2020. The copyright holder for this preprint (which was not certified by peer review) is the author/funder, who has granted medRxiv a license to display the preprint in perpetuity.

It is made available under a CC-BY 4.0 International license .

Supplementary Materials

medRxiv preprint doi: https://doi.org/10.1101/2020.11.19.20234229; this version posted November 22, 2020. The copyright holder for this preprint (which was not certified by peer review) is the author/funder, who has granted medRxiv a license to display the preprint in perpetuity.

It is made available under a CC-BY 4.0 International license .

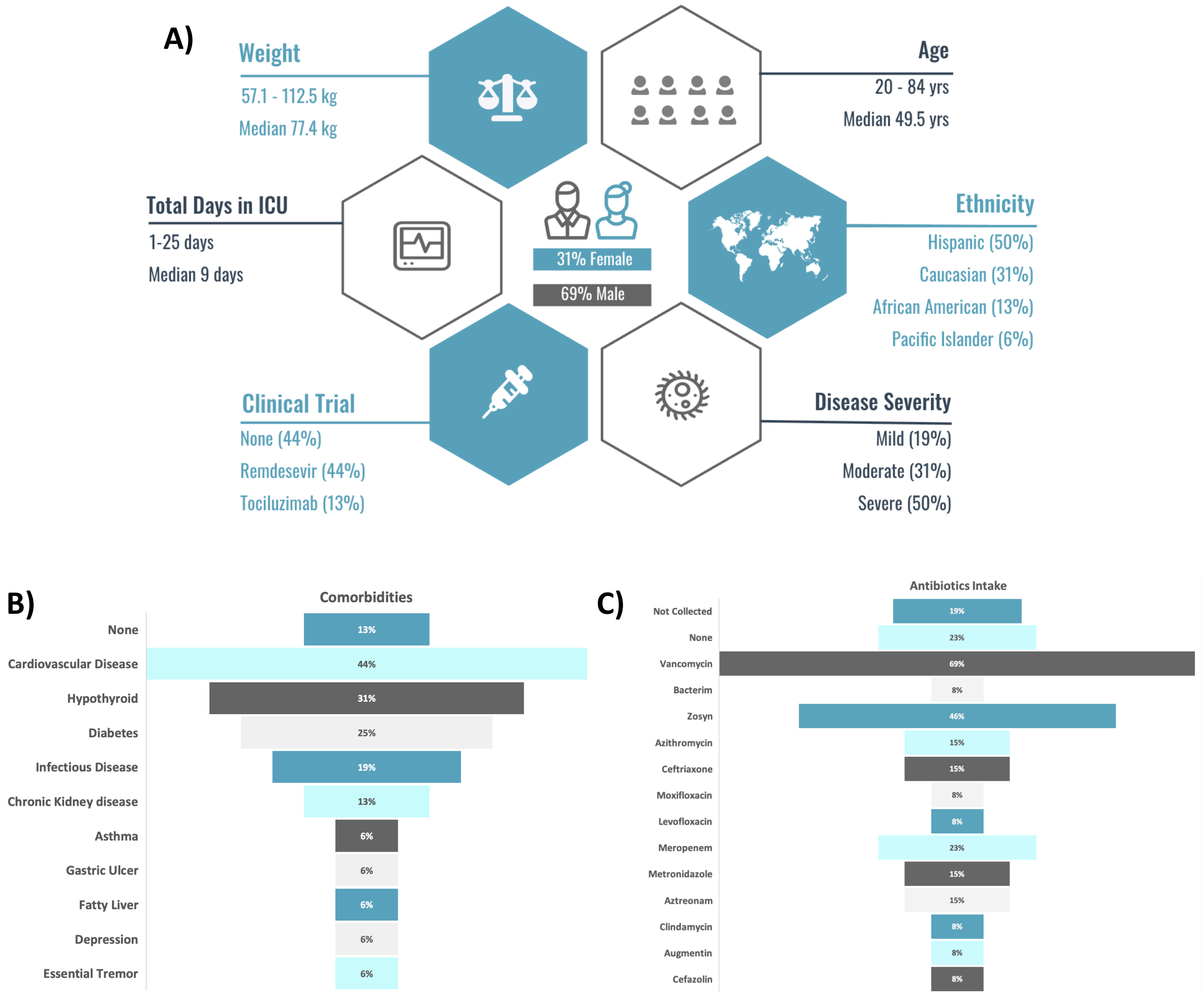

Figure S1. Patient (n=16) demographics (A), antibiotics intake (B), comorbidities (C).

medRxiv preprint doi: https://doi.org/10.1101/2020.11.19.20234229; this version posted November 22, 2020. The copyright holder for this preprint (which was not certified by peer review) is the author/funder, who has granted medRxiv a license to display the preprint in perpetuity.

It is made available under a CC-BY 4.0 International license .

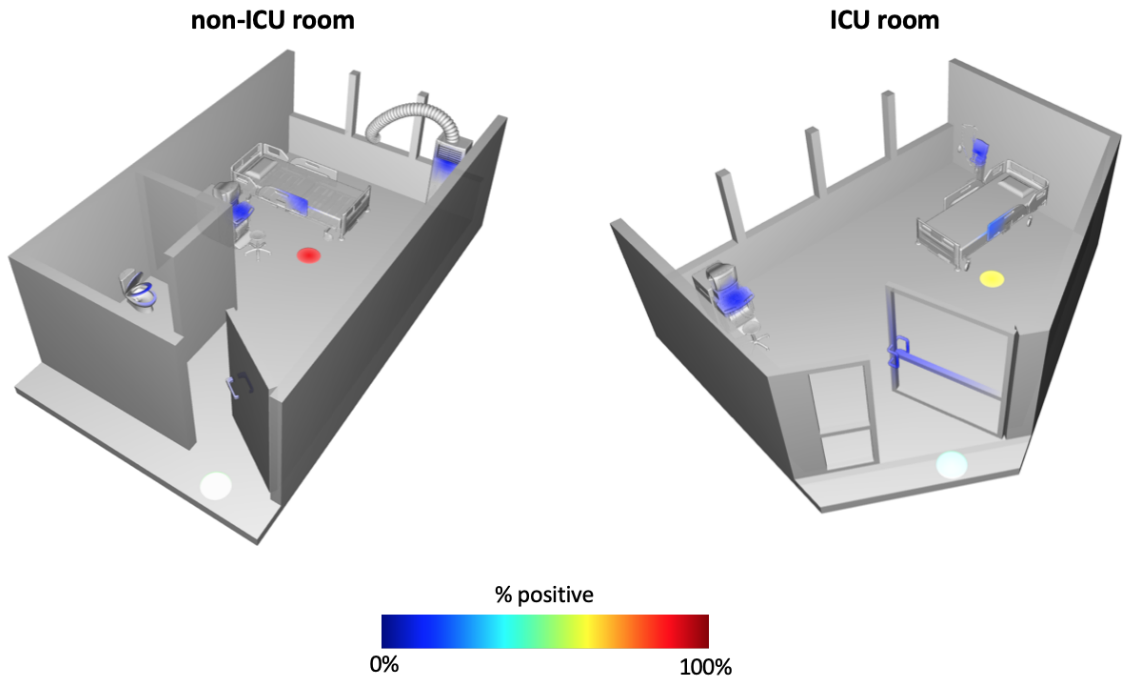

Figure S2. Ili' spatial mapping of standard hospital (non-ICU) room and intensive care unit (ICU) room. Heatmap depicts the percent of samples collected at each site that were positive for SARS-CoV-2.

medRxiv preprint doi: https://doi.org/10.1101/2020.11.19.20234229; this version posted November 22, 2020. The copyright holder for this preprint (which was not certified by peer review) is the author/funder, who has granted medRxiv a license to display the preprint in perpetuity.

It is made available under a CC-BY 4.0 International license .

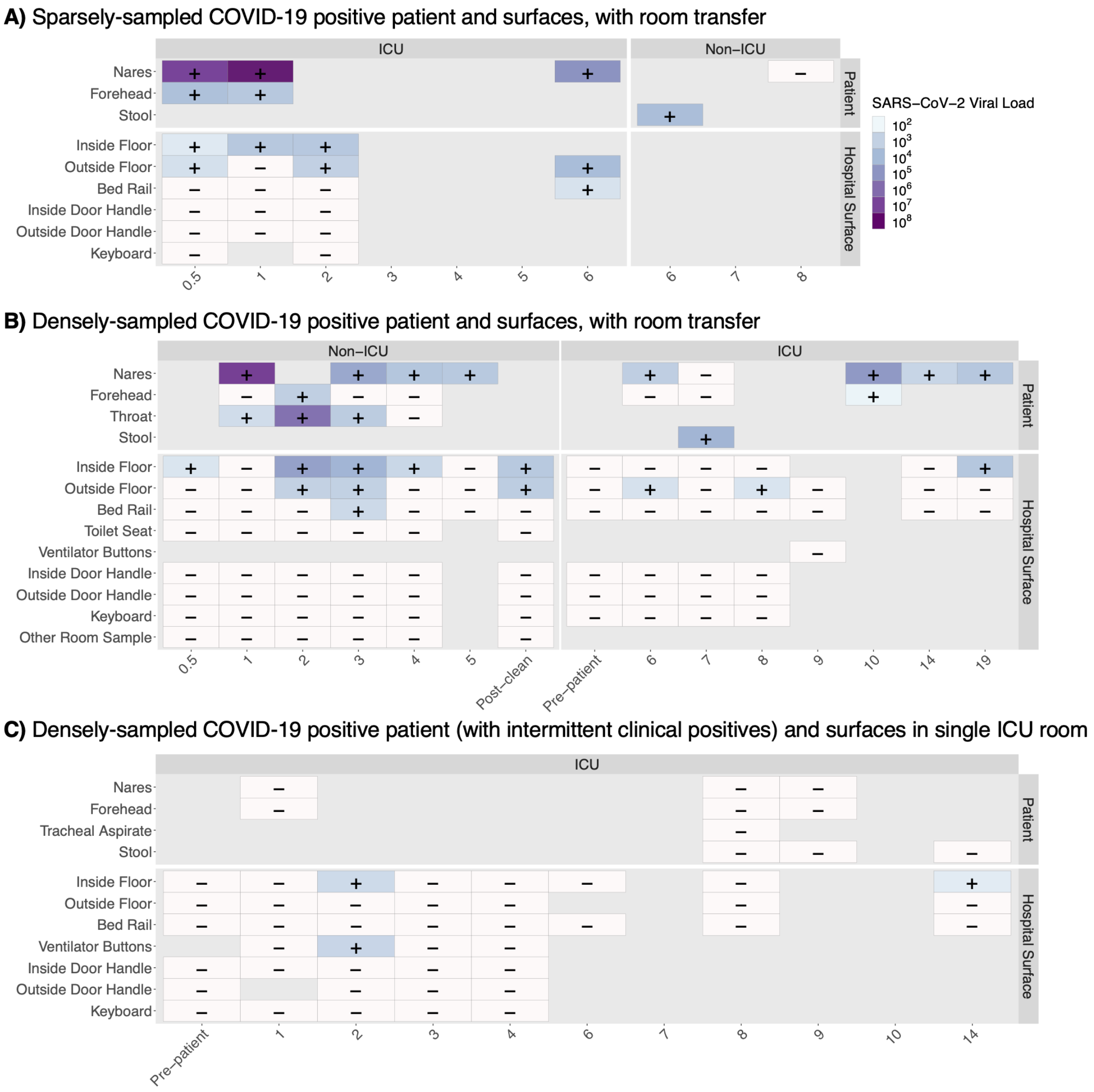

Figure S3. Snapshot of variability in longitudinal sample collection and SARS-CoV-2 viral load per swab between patients and their hospital rooms, starting at patient admission time. For samples where SARS-CoV-2 was detected (+), a darker color indicates a higher viral load. White boxes represent samples with no detectable virus (-). Patient **A** was admitted 12 days after symptom onset and was moved to a general surgery unit room after 6 days in the ICU. Patient **B** was admitted 8 days after symptom onset and moved from general surgery to the ICU, where they were intubated. Patient **C** was admitted to the ICU 9 days after symptom onset, and despite having symptoms

medRxiv preprint doi: https://doi.org/10.1101/2020.11.19.20234229; this version posted November 22, 2020. The copyright holder for this preprint (which was not certified by peer review) is the author/funder, who has granted medRxiv a license to display the preprint in perpetuity.
It is made available under a CC-BY 4.0 International license .

consistent with COVID-19 repeatedly tested negative by clinical nasopharyngeal swab; their only clinical positive came from a tracheal aspirate sample mid-way through their stay in the ICU.

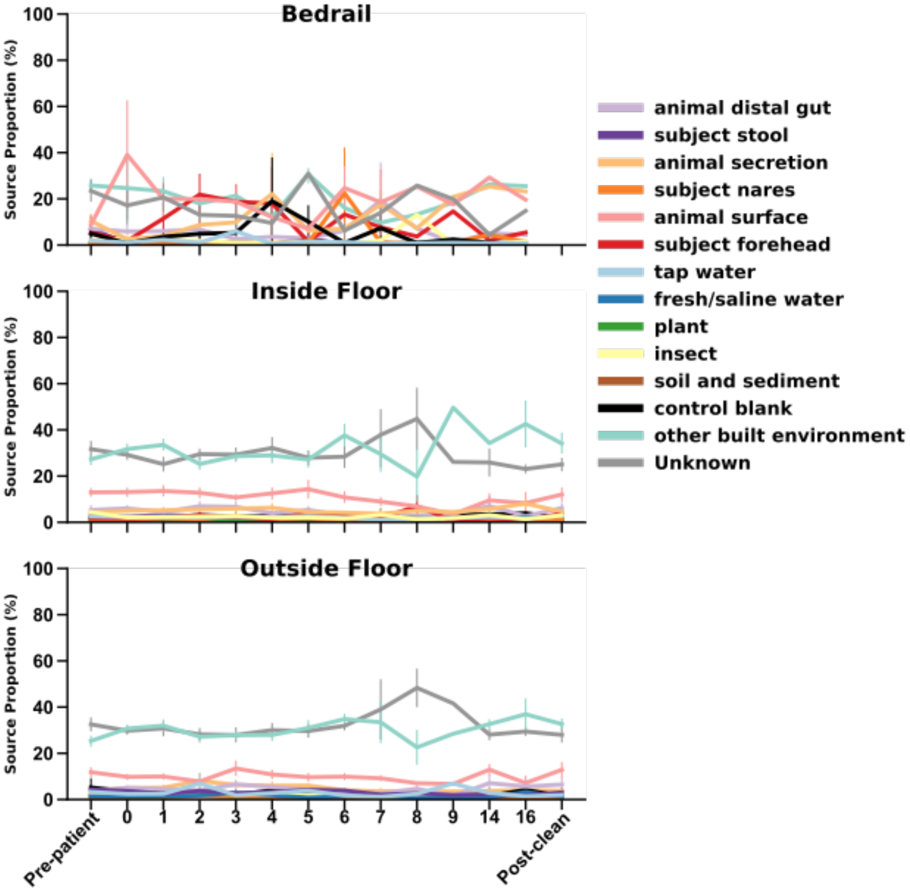

Figure S4. Source tracker on meta-analysis data. Floor samples formed a distinct cluster in this dataset; source tracking *(31)* with floor samples (n=215) as the sink and meta-analysis samples (n=1,990) as the source reveals that these floor samples match other built environment samples. The other built environment samples included in this meta-analysis were mostly floor (27.7%), faucet handles (19.6%), and gloves (15%).

medRxiv preprint doi: https://doi.org/10.1101/2020.11.19.20234229; this version posted November 22, 2020. The copyright holder for this preprint (which was not certified by peer review) is the author/funder, who has granted medRxiv a license to display the preprint in perpetuity.

It is made available under a CC-BY 4.0 International license .

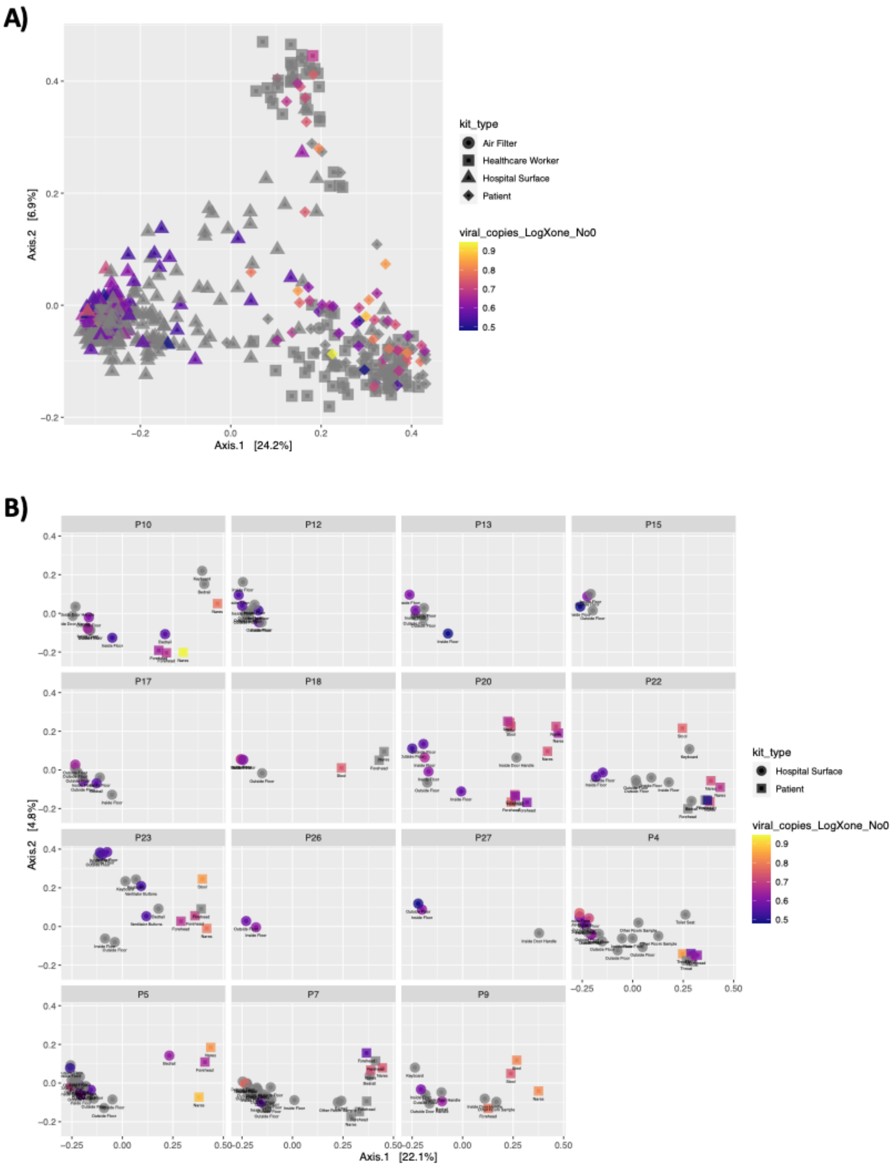

Figure S5. Beta-diversity has a statistically significant but weak correlation with viral load. PCoA of unweighted UniFrac distances between samples, with SARS-CoV-2 positive samples colored by viral load across the whole dataset (**A**) and subset by each patient with at least one surface positive (**B**). Statistical analysis performed with Adonis (PERMANOVA) found a small ($R^2 < 0.01$) but significant (*p-value* = 0.043) association between beta-diversity and viral load across all samples.

medRxiv preprint doi: https://doi.org/10.1101/2020.11.19.20234229; this version posted November 22, 2020. The copyright holder for this preprint (which was not certified by peer review) is the author/funder, who has granted medRxiv a license to display the preprint in perpetuity.

It is made available under a CC-BY 4.0 International license .

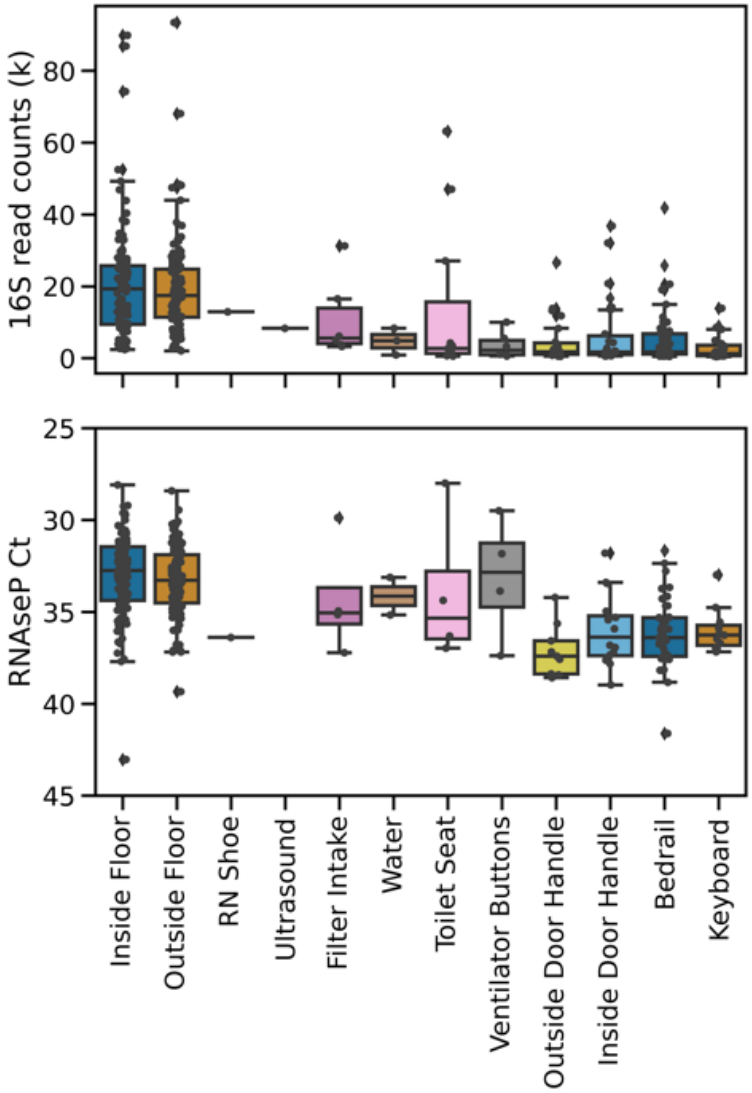

Figure S6. Bacterial (16S rRNA gene amplicon sequencing read count) and human biomass (RNAse P Ct) is higher in floor samples than other surface sample types.

medRxiv preprint doi: https://doi.org/10.1101/2020.11.19.20234229; this version posted November 22, 2020. The copyright holder for this preprint (which was not certified by peer review) is the author/funder, who has granted medRxiv a license to display the preprint in perpetuity.

It is made available under a CC-BY 4.0 International license .

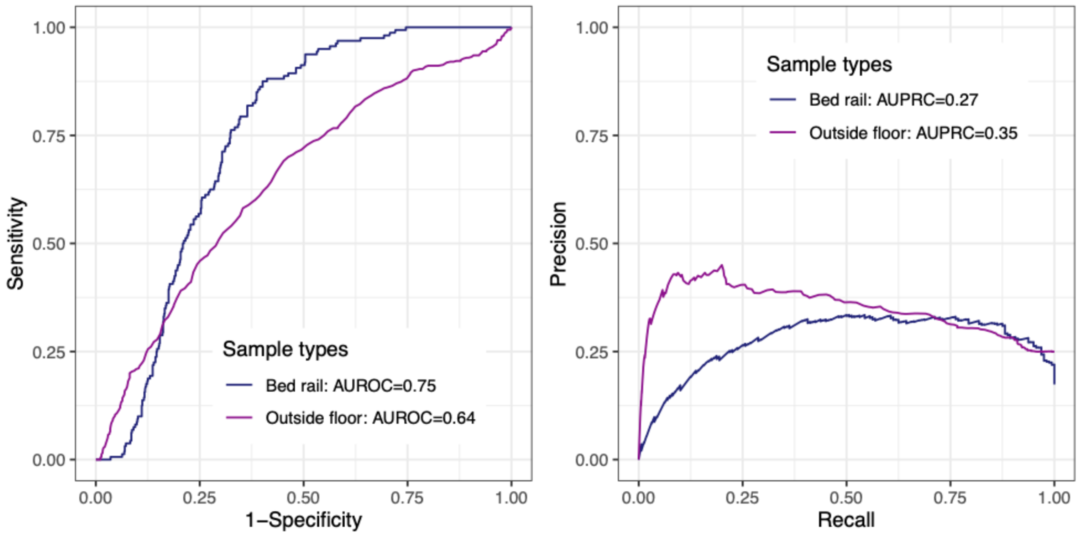

Figure S7. Random Forest classifier performance with 100-fold cross validation in the outside floor (n=108; 81 not detected vs. 27 positives) and bed rail samples (n=46; 38 not detected vs. 8 positives).

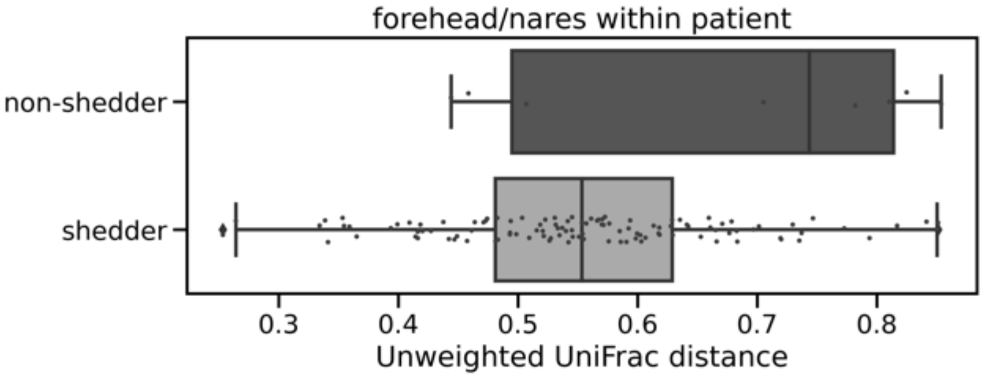

Figure S8. Unweighted UniFrac distance between forehead and nares samples from the same host. ‘Shedder’ (n=12) is a patient who had detectable virus on the surface in their room and ‘non-shedder’ (n=4) did not. Bootstrapped Kruskal-Wallis p-value is 0.003.

medRxiv preprint doi: https://doi.org/10.1101/2020.11.19.20234229; this version posted November 22, 2020. The copyright holder for this preprint (which was not certified by peer review) is the author/funder, who has granted medRxiv a license to display the preprint in perpetuity.

It is made available under a CC-BY 4.0 International license .

Table S1. Hospital surface materials and cleaning practices.

Surface	Material	Cleaning schedule	Cleaning material	Who touches
Inside floor	Vinyl tile	Variable, infrequent (~1/week)	Bleach	Universal – health care workers, visitors, patient if ambulatory
Outside floor	Vinyl tile	Variable, infrequent (~1/week)	Bleach	Universal – health care workers, visitors, patient if ambulatory
Inside door handle	Plastic in ICU; Steel outside ICU	Variable, infrequent (~1/week)	Hydrogen peroxide wipes	Universal – health care workers, visitors
Outside door handle	Plastic in ICU; Steel outside ICU	Variable, infrequent (~1/week)	Hydrogen peroxide wipes	Universal – health care workers, visitors
Bed Rail	Plastic	Variable, health care workers wipe down intermittently typically once at the start of shift (~2x daily)	Hydrogen peroxide wipes	health care workers, patient
Keyboard	Plastic	Variable, health care workers wipe down intermittently typically once at the start of shift (~2x daily)	Hydrogen peroxide wipes	health care workers
Air vent intake	Plastic	Variable, infrequent (~1/week)	Hydrogen peroxide wipes	health care workers
Ventilator buttons	Plastic	Variable, will be wiped down after no longer needed by patient (average 2-3 times a week)	Hydrogen peroxide wipes	health care workers (specifically respiratory therapists, MD)
Toilet seat	Ceramic	Variable, at least deep cleaned after patient discharged (average 2-3 times a week)	Bleach	Patient, visitors

Data file S1. Statistical analysis of pairwise differences in log-ratio across sample types from figure 3D trajectory plot.

Data file S2. Top 100 random forest importance ranks and GreenGenes taxonomy from nares, forehead, stool, and inside floor samples.
